# Hypercoagulable state and effect of low-molecular-weight heparin prophylaxis on coagulation after lung cancer resection: results from thrombo-elastography

**DOI:** 10.1007/s11748-024-02062-6

**Published:** 2024-07-26

**Authors:** Xiaoxiao Yang, Yongsheng Cai, Lihui Ke, Bo Wei

**Affiliations:** Department of Thoracic Surgery, Beijing Tiantan Hospital Affiliated to Capital Medical University, Beijing, China

**Keywords:** Thrombo-elastography, Lung cancer, Hypercoagulability, Low-molecular-weight heparin, Venous thromboembolism

## Abstract

**Background:**

Lung cancer patients undergoing surgery are at increased risk for Venous thromboembolism (VTE). We monitored changes in perioperative coagulation status through Thrombo-elastography (TEG), and monitored the anticoagulant effect of low molecular weight heparin through TEG for the first time.

**Methods:**

From July 2019 to January 2020, 207 patients receiving curative surgery were retrospectively screened. and 23 patients were excluded because they did not meet the inclusion criteria. Blood samples were required at three time points (prior to, the first and third day after surgery). Some patients were administrated nadroparin calcium daily from the first day after surgery. Repeated measures ANOVA and Chi-square test were used to analyze the coagulation states variation. To balance the confounders, propensity score matching (PSM) was used to determine the differences of coagulation states between patients with or without Low-molecular-weight heparin (LMWH) prophylaxis.

**Results:**

In 184 patients, TEG parameters displayed significant procoagulant changes after lung surgery but conventional coagulation tests exhibited paradoxical trends. There were 6.5% (12/184) of patients identified as hypercoagulability before surgery. According to TEG results, the proportion of patients with hypercoagulability rose from 21.7% to 25% postoperatively, but more were classified into platelet or mixed hypercoagulability at third day compared with that at first day (3.8% vs 14.1%, *P* < 0.001). By PSM analysis, there were no significant differences in the proportion of hypercoagulable patients postoperatively between chemoprophylactic and nonprophylactic group.

**Conclusions:**

TEG was eligible to distinguish changing states of hypercoagulability postoperatively and indicate the role of platelet in blood hypercoagulability. Administration of postoperative LMWH prophylaxis showed little mitigation on hypercoagulable states.

**Supplementary Information:**

The online version contains supplementary material available at 10.1007/s11748-024-02062-6.

## Introduction

Venous thromboembolism (VTE), which consists of deep vein thrombosis (DVT) and pulmonary embolism (PE), presents as a major life-threatening complication that contributes to increasing morbidity and mortality [1]. Patients undergoing oncological surgery were considered to be at high risk of postoperative VTE [2]. Malignant patients often experienced considerable thromboembolic disorders and procoagulant changes following surgical operation [3], which is believed to facilitate the formation of venous thrombi [4]. Therefore, surveillance of coagulation state during perioperative phrases are necessary for overall acknowledgement of patient’s hemostatic change and dynamics in thrombotic risk, which is beneficial for improving healthcare of this complication.

Thromboglothiography (TEG) was originally invented by Hartert and is described as a sensitive blood coagulation test that analyzes the kinetics of clot formation, from the initial fibrin thread to fibrinolysis. TEG is used to assess the overall coagulation kinetics and strength of whole blood and has been successfully used clinically to detect hypercoagulable states [5–7]. It takes the coagulation factors, fibrinogen and platelet into account in the process of natural coagulation in vitro. This test minimizes the impact of shortage in dynamic interaction of various procoagulant factors (platelets especially) in clot formation, which is the inherent defect in conventional coagulation tests that introduce only plasma-based specimen. As a viscoelastic method, TEG results unscramble the real-time functional state of patients’ coagulation and fibrinolysis system, and has been used in assessing hypercoagulability or impaired coagulability in many circumstances like pregnancy, sepsis, severe injury [8]. Additionally, there is a potential application of TEG in assessing hypercoagulation following surgery [9].

Low-molecular-weight heparin (LMWH) is recommended as standard chemoprophylaxis for patients undergoing cancer surgery, but the utilization of proper prophylactic strategy was underperformed, including insufficient administrative dose and lack of extended chemoprophylaxis in China, which was considered to be due to inappropriate recognition of high-risk patients and scruple about bleeding risk caused by anticoagulation [10]. Even LMWH was recognized to reduce the risk of postoperative VTE, it was found that patients receiving LMWH thromboprophylaxis still develop VTE [11]. A randomized study, that compared the prophylactic effect of high- and low-dose LMWH in patients undergoing lung surgery, showed that even a high dose administration did not provide adequate prophylactic effect [12]. On the other hand, a new meta-analysis indicated that in patients with lung cancer, the reduction of VTE risk rendered by LMWH was accompanied by an increasement in bleeding risk at the meantime [13]. Therefore, monitoring the effectiveness of postoperative prophylactic LMWH is necessary for individualized chemoprophylaxis appropriate for patients with different VTE risk stratification to optimize the outcomes. Currently, there were no formal recommendations of monitoring solutions. Anti-Xa activity and prothrombin fragment F1 + 2 have been used in monitoring the dosage of LMWH [14], but these assays are not routinely tested in clinical practice and displayed inconsistent reliability. TEG, as a rapid and cost-efficient test, has been implemented in cardiovascular surgery and proved to facilitate improved individual anticoagulation management [15, 16]. Previous study showed that enoxaparin sodium exhibited a dose-dependent inhibition of clot formation on TEG tracing [17]. Thus, we assumed that the potency of TEG in monitoring the effects of LMWH on blood coagulation for lung cancer surgery could be highlighted.

In this study, we aimed to figure out how the coagulation profile changes after curative surgery in lung cancer patients by means of TEG, and to investigate the influence of postoperative LMWH administration on coagulation status for thrombophylactic purpose.

## Materials and methods

### Population and material

In this study, patients with primary lung cancer hospitalized for surgery were retrospectively studied from July 2019 to January 2020. The exclusion criteria listed as (1) any anticoagulation or anti-platelet therapy within 30 days before surgery; (2) malignancy history or neoadjuvant chemotherapy/radiotherapy before surgery; (3) VTE event prior to surgery; (4) lack of necessary data. All eligible patients received radical surgery under general anesthesia. Venous ultrasound of lower limbs was performed postoperatively in order to detect DVT, and patients suspected of PE with clinical symptoms were arranged to computer tomography pulmonary angiography (CTPA). Patients given postoperative VTE chemoprophylaxis received subcutaneous nadroparin calcium (10,250 WHO Unit/mL) every night from the first day postoperative until discharge and the dose was calculated according to weight, ranging from 0.3 mL to 0.6 mL in our practice. All patients were given gradient compress stocking during operation and early days after surgery until they were allowed to off-bed activity.

Blood tests at three time points were needed (baseline prior to surgery, the first and third day postoperatively) for all included patients. The clinical data (age, gender, body mass index, smoking history, chronical disease including hypertension, coronary heart disease, diabetes mellitus, chronical pulmonary disease, surgical information, histological type, anticoagulation information, VTE event) and blood tests results were obtained from electronical medical database. The pathological stage of lung cancer was determined according to 8th edition IASLC classification of TNM staging [18]. All patients were followed until discharge. The study was conducted in accordance with the Declaration of Helsinki (as revised in 2000). Patient consents were waived because of the retrospective nature. This research has been approved by hospital ethical commitment board (KY2023-111-02).

### Laboratory measurement

Blood samples were collected via venipuncture in forearm and were delivered to automated analysis in the laboratory within 1 h after sampling. For patients receiving LMWH, the blood sample was obtained after subcutaneous injection of nadroparin calcium. TEG assay was performed on citrated sample by the TEG® 5000 Thromboelastograph® Hemostasis Analyzer System activated by kaolin and 20 μL of calcium chloride. We collected TEG parameters including reaction time (R, the time from the onset of test to the first detectable formation of clot), kinetics (K, the time from the clotting to the formation at certain strength level of 20 mm in amplitude), α angle (the angle between the baseline and the imaginary tangent line to the curve), maximum amplitude (MA, maximal amplitude of the TEG curve), and other conventional coagulation tests including international normalized ratio (INR), fibrinogen, platelet, D-dimer concentration.

The hypercoagulative state was categorized into three types according to the manufacturer: (1) enzymatic hypercoagulability, R < 5 min or α angle > 72° or K < 1 min, and MA ≤ 70 mm; (2) platelet hypercoagulability, R ≥ 5 min, α angle ≤ 72°, K ≥ 1 min, and MA > 70 mm; (3) mixed hypercoagulability, R < 5 min or α angle > 72° or K < 1 min, and MA > 70 mm.

### Statistical methods

The data were expressed as mean ± standard derivation, median with interquartile range, or ratio according to their statistical distribution. Repeated measures one-way ANOVA to detect the variation of laboratory measurements perioperatively, and if sphericity was not assumed, the results were then modified by Greenhouse–geisser method. Spearman’ s correlation was applied to test correlation between TEG and conventional coagulation parameters. Chi-square test and Fisher’s exact test were performed accordingly to compare the different proportion of three types of hypercoagulability at three time points and to determine the relationship between hypercoagulative state and anticoagulation therapy. For the purpose to balance the distribution of patient characteristic (age, sex, pathological staging, postoperative hospital stay) and surgical procedure (surgical approach, surgical time), we used propensity score matching (PSM) with a caliper less than 0.02 to select patients from those without postoperative LMWH prophylaxis to match those receiving prophylaxis. The propensity score was calculated from logistic regression model incorporating potential confounders. As a reminder, patients with therapeutic administration of LMWH after established VTE were beforehand excluded. Two-tailed *P*-value < 0.05 of each test was considered statistically significant throughout the study. All data were statistically processed using IBM SPSS statistics version 26.0 (Armonk, NY: IBM Corp).

## Results

### Population characteristics

From July 2019 to January 2020, a total of 207 patients were screened. Twenty-three patients did not meet our inclusion criteria and were excluded: (1) Seven patients received anticoagulant or antiplatelet therapy within 30 days before surgery; (2) Three patients had a history of malignancy or neoadjuvant chemotherapy/radiotherapy before surgery; (3) Five patients had venous thromboembolism before surgery; (4) 8 patients lacked necessary data. After exclusion, 184 eligible patients were finally enrolled for analysis (Table 1). The average age of patients when admitted into hospital was 58.7 ± 9.7, and 39.7% of the population was male. Most patients were taken to video-assisted thoracoscopic surgery (VATS) and the other 18 (9.8%) patients undergone thoracotomy. Lobectomy and sublobar resection were considered as most common surgical procedure and only 3 (1.6%) patients received pneumonectomy. In terms of pathological type, adenocarcinoma (87.0%) accounted for the major type of lung cancer, and 10 (9.8%) patients were diagnosed as squamous cell carcinoma. Until discharge, 11 patients (6.0%) were found VTE, and all these VTE events were DVT.

**Table 1:** Clinical characteristics of patients (*n* = 184)

Characteristics	
Age (years)	58.7 ± 9.7
Gender (male)	73 (39.7)
BMI (kg/m^2^)	24.3 ± 3.5
Smoking history	47 (25.5)
Hypertension	71 (38.6)
Coronary heart disease	17 (9.2)
Diabetes mellitus	20 (10.9)
Chronical pulmonary disease	12 (6.5)
*Surgical approach*	
Thoracotomy	18 (9.8)
VATS	166 (90.2)
*Surgery*	
Sublobar resection	66 (35.9)
Lobectomy	115 (62.5)
Pneumonectomy	3 (1.6)
Duration of operation (mins)	127.0 ± 46.5
Intraoperative blood loss (ml)	50 (50–100)
Postoperative hospitalization (days)	5 (4–7)
Postoperative anticoagulation	46 (25.0)
*Histological type*	
Adenocarcinoma	160 (87.0)
Squamous cell carcinoma	18 (9.8)
Others	6 (3.3)
*TNM stage*	
0	43 (23.4)
I	100 (54.3)
II	14 (7.6)
III	27 (14.7)
VTE event	11 (6.0)
DVT	11 (6.0)
PE	0 (0)
LWMH prophylaxis	37(20.1)

Measurement data are expressed as mean ± standard deviation (SD) and median (interquartile range) according to their distribution; Enumeration data are expressed as number (%)

*BMI* body mass index, *VATS* video-assisted thoracoscopic surgery, *VTE* venous thromboembolism, *DVT* deep vein thrombosis, *PE* pulmonary embolism, *LWMH* Low-molecular-weight heparin

### Variation of perioperative laboratory results

**Fig. 1:**
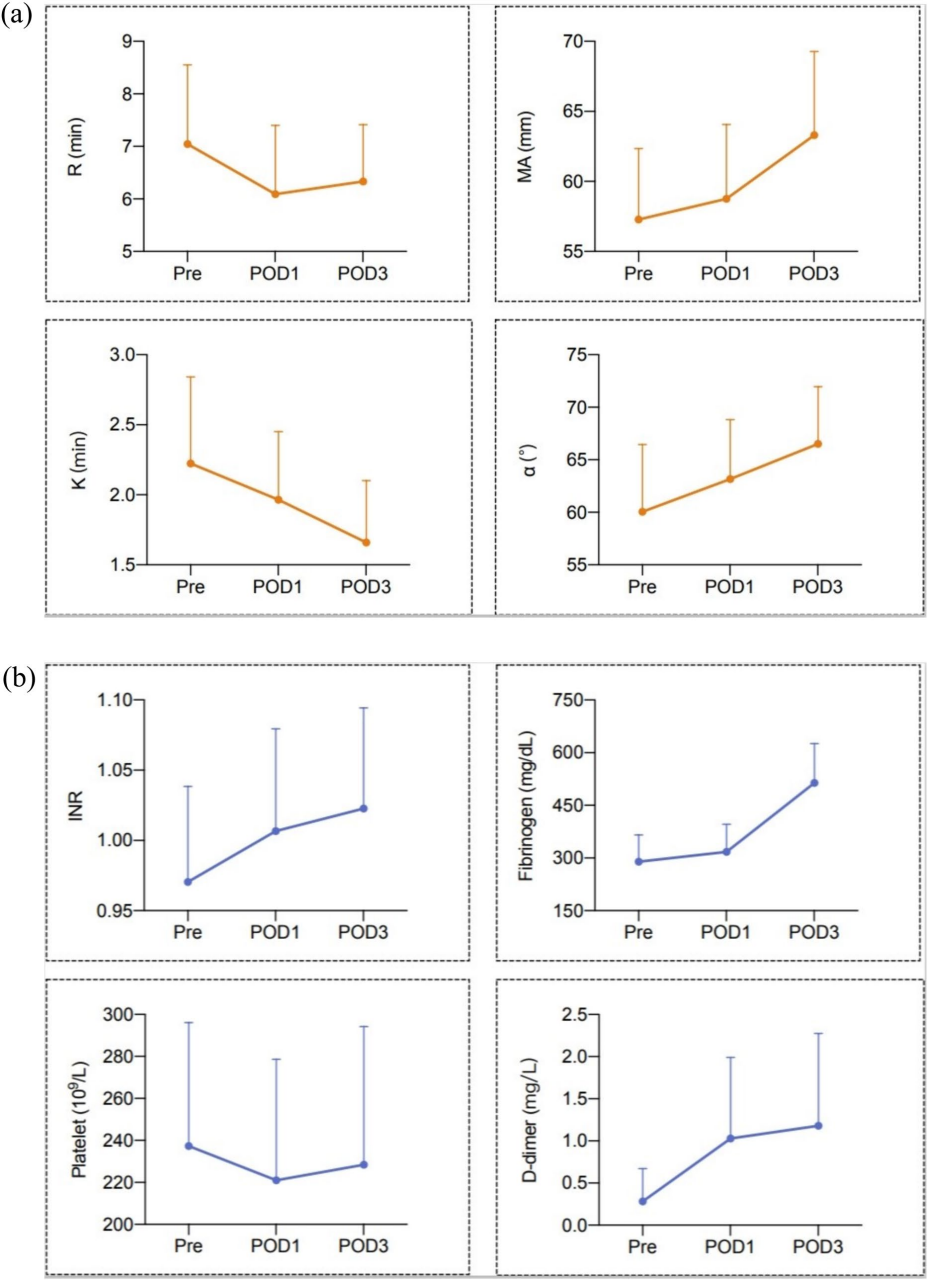
The variation of paraments of thrombo-elastography (**a**) and conventional coagulation tests (**b**) at three time points (*Pre* preoperative, *POD1* postoperative day 1, *POD3* postoperative day 3). *MA* maximal amplitude, *INR* International normalized ratio

In whole population, the ANOVA analysis illustrated significant changes compared to preoperative baseline for all parameters (Fig. 1). In terms of TEG variables, the decrease of R and K as well as the increase of MA and α angle indicated a tendency toward hypercoagulable state after surgery, while the conventional coagulation tests showed paradoxical trends, in which the hypo-coagulation was indicated by the INR dropping and hypercoagulation by fibrinogen and D-dimer elevating.

### Correlations of measures of coagulation

Analysis revealed some significant correlation between TEG and conventional measures of hemostasis (Table 2). The fibrinogen and platelet were significantly correlated with MA (*r* = 0.44–0.51, *P* < 0.05), and other correlations were deemed as trivial and insignificant.

**Table 2:** Correlation (r) between TEG parameters and conventional coagulation tests

TEG parameters	Conventional coagulation tests			
	INR	Fibrinogen	Platelet	D-dimer
R	0.02–0.07	0.13–0.28	0.04–0.10	0.07–0.19
MA	0.01–0.10	0.26–0.44	0.36–0.51	0.07–0.14
α	0.05–0.06	0.08–0.14	0.21–0.29	0.08–0.26
K	0.067–0.12	0.05–0.10	0.18–0.30	0.04–0.21

*INR* International normalized ratio, *MA* maximal amplitude

### Changes of perioperative coagulation status

**Fig. 2:**
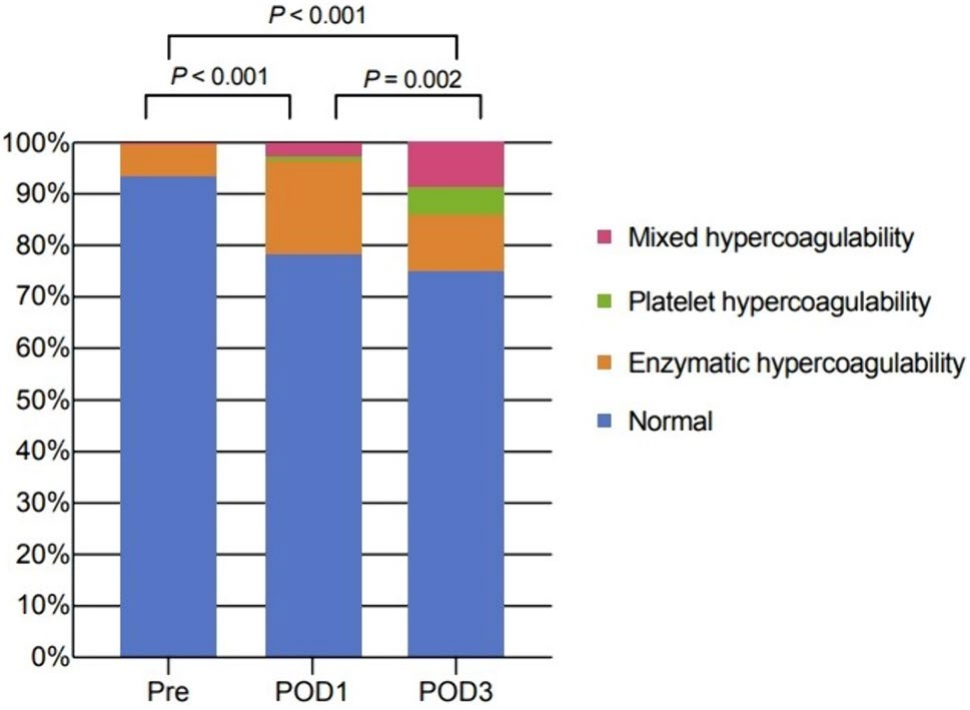
Proportion of different states of hypercoagulability at three time points (*Pre* preoperative, *POD1* postoperative day 1, *POD3* postoperative day 3)

The baseline status of coagulation of whole population before surgery was virtually within normal range indicated by TEG (Fig. 2). Only 12 (6.5%) were classified as hypercoagulability, 11 of whom were enzymatic hypercoagulability. At the first day after surgery, the proportion of patients with hypercoagulable states significantly increased compared to baseline (21.7% vs 6.5%, *P* < 0.001), but most of them (33/40) were still classified into enzymatic hypercoagulability according to TEG references. At the third day, patients with hypercoagulable states took up 25% of the whole population and 14.1% were platelet or mixed hypercoagulability where platelet played an essential role.

### Differences in coagulation status between chemoprophylaxis and no-chemoprophylaxis group

**Fig. 3:**
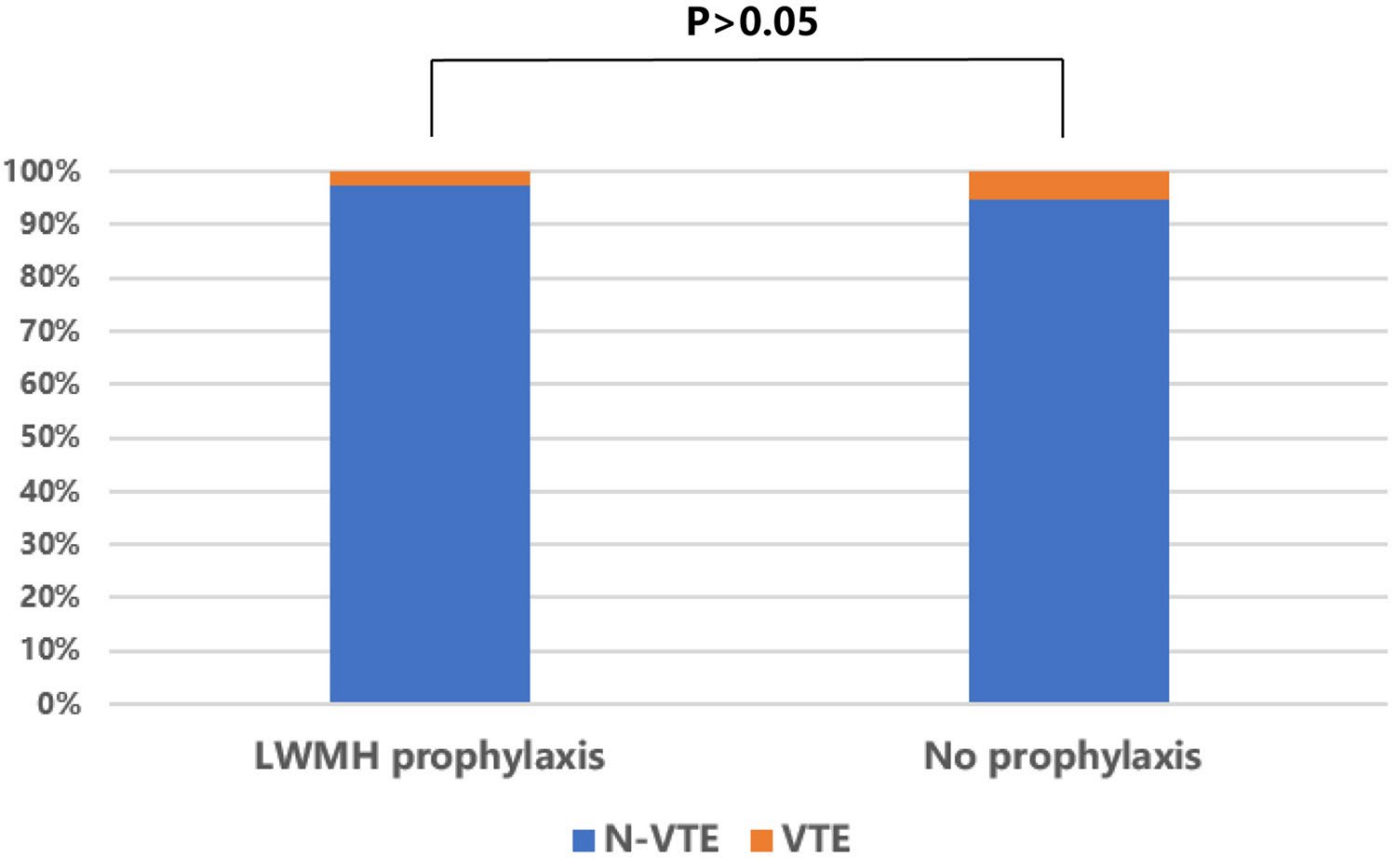
Comparison of VTE incidence between those who received LWMH and those who did not receive LWMH after PSM. *VTE* Venous thromboembolism, *LWMH* Low-molecular-weight heparin (LMWH), *PSM* propensity score matching

The PSM analysis selected 37 pairs of patients (Table 3) with balanced baseline clinical characteristics (Supplement 1). After PSM, 1 of 37 patients (2.7%) who received LWMH developed VTE, whereas 2 of 37 patients (5.4%) who did not receive LWMH developed VTE. There was no statistically significant difference between the two groups (Fig. 3).The coagulation state was identical before surgery between two groups. At the first day after surgery, 24.3% patients (*n* = 9) receiving LWMH prophylaxis were identified as hypercoagulability, which was significantly higher compared to that before surgery, and the number is 10 at the third day postoperatively. In patients without prophylaxis, 6 patients were found hypercoagulability at first day and the number doubled at third day. But the difference in the proportion of hypercoagulable patients between two groups reached no statistical significance at both time points postoperatively (*P* = 0.386; *P* = 0.611, respectively).

**Table 3:** The proportion of patients in different coagulation states with and without chemoprophylaxis

	LWMH prophylaxis (*n* = 37)		No prophylaxis (n = 37)		*P*-value
	Hypercoagulation	Normal	Hypercoagulation	Normal	
Pre	2 (5.4)	35 (94.6)	2 (5.4)	35 (94.6)	1.000
POD1	9 (24.3)	28 (75.7)	6 (16.2)	31 (83.8)	0.386
POD3	10 (27.0)	27 (73.0)	12 (32.4)	25 (67.6)	0.611

Propensity score matching selected 37 pairs of patients receiving LWMH prophylaxis and no prophylaxis. Data was expressed as *n* (%). *DVT* deep vein thrombosis, *LWMH* low-molecular-weight heparin, *Pre* preoperative, *POD1* postoperative day 1, *POD3* postoperative day 3

## Discussion

This study revealed a hypercoagulative trend among patients after lung cancer resection and demonstrated that there were significantly diverse states of hypercoagulability following surgery by means of TEG measurement, in which the proportion of platelet-dominated hypercoagulability prevailed gradually after surgery. PSM analysis indicated that administration of LMWH prophylaxis was not associated with significant mitigation in patient procoagulant state after surgery.

Conventional coagulation tests have been used for surveillance for decades but manifesting suboptimal ability in revealing coagulation function for surgery patients [9, 19]. In this study, all incorporated TEG parameters mutually verified that there was a procoagulant tendency after lung cancer surgery, but conventional coagulation tests exhibited paradoxical changes. Mao and colleagues performed TEG and conventional coagulation tests on patients with DVT and healthy controls, and they discovered that a hypercoagulable tendency was found by TEG not by conventional coagulation tests like APTT, PT or platelet [20]. In our study, INR, which is the calculation generated from the PT, also showed undesired ability to indicate postoperative hypercoagulative status. Consistently, another research also proved that PT and APTT were underpowered in revealing significant hypercoagulable state [21]. INR tests are performed in platelet-free blood sample, which means it is ineligible to vindicate the role of cell surface in the coagulation course. INR is in alignment with fibrin generation, reflecting the number and function of particular coagulation factors (prothrombin, FV, FVII and FX), therefore, the dropping of INR in early days of post-operation may associate with the consumption of coagulation factors during surgery. However, it is not necessary to show the hypocoagulative state given that INR represent incomplete measurement of clot formation [5].

Contrary, TEG can unfold more information about the phases of clot formation based on whole blood, including activation of coagulation cascade, thrombin generation, platelet activation and aggregation [18]. TEG was able to identify hypercoagulability in colorectal and prostate cancer [9, 22]. As for lung cancer, Davies et al. found that some parameters in rotational thromboelastometry (ROTEM) that is similar to TEG test were significantly different in lung cancer patients compared to health controls [23]. Nevertheless, these researches defined hypercoagulation by using few parameters and had not assessed the dynamic changes of these parameters, which underutilized of TEG in assessing overall aspects of blood coagulation status. In our study, individual coagulation state and the defined hypercoagulability were set down by incorporating a wide panel of TEG parameters according to their normal range, which provided full-scale assessment of coagulation conditions and was more applicable in clinical practice.

It is very necessary to emphasize that the VTE risk differs significantly among individuals, also, the risk profile can be dynamically changed within the same patient over time. The surveillance the changes of coagulation state of patients undergoing surgery can shed insight into surgery-induced coagulation system fluctuation, promoting the understanding of the true course of thrombosis complication [24]. Surgical operation introduces traumatic stress but removes the tumor load, which represents a shortened impact on patient hematologic system instead of a prolonged effect that is more common in multi-period chemotherapy, so the risk of VTE seems to predominantly occur within the initial postoperative time [25]. Notably, the bleeding risk at the early period after surgery can be simultaneously higher, which may baffle the healthcare management in balancing the VTE risk and bleeding risk, so elaborate surveillances of coagulation state during the limited time widow of hypercoagulability should be prioritized.

According to the classification of TEG reference, the hypercoagulable state was divided into three different types, including enzymatic, platelet and mixed hypercoagulability. At the early days after surgery, the proportion of patients with platelet or mixed hypercoagulability raised significantly from 3.8% to 14.1%, and both hypercoagulable states meant that MA was higher than upper limit. At the same time, MA was significantly elevated compared to baseline. MA reflects the strength of clot that depends on platelet concentration and function as well as platelet-fibrinogen cross-link in the clot formation. As shown in this study, platelet displayed a decent correlation with MA. Platelet concentration exhibited a trend that was downside at the first day and rebounded at the third day postoperatively, which corresponded to the alteration of distribution in hypercoagulability. Apart from quantifying concentration to justify the role of platelet in the coagulation process, the contribution of platelet to procoagulant state can be more validated by their hyperactivation in the hemostasis process [26]. Study utilizing ROTEM suggested that testing array incorporating platelet contribution are more recommendable to detect hypercoagulation [27]. Similarly, a higher MA in TEG tracing is associated with enhanced platelets adherence and aggregation that lead to intensive strength of clot formation. These results suggested that in the early period, activated platelet played an important role in postoperative hypercoagulative state among lung cancer patients.

Although the pathophysiologic theory underlying the prothrombotic state for cancer patients remains unsolved, several mechanisms concerning platelets activation have been proposed and there are growing recognition of platelet contribution to cancer associated venous thrombosis [28]. Cancer cell can initiate the coagulation cascade by upgrading the secretion of tissue factor (TF), which leads to thrombin generation and platelets activation [29]. Cancer cell can also directly stimulate platelets by releasing platelet activation receptor or providing binding site for platelet aggregation [30]. In lung cancer patients receiving chemotherapy, platelets had enhanced phosphatidylserine exposure and elevated secretion of platelet-derived microparticle compared to health controls, which suggested that platelets obtained increased procoagulant activity after chemotherapy [31]. Surgery also represents as an important driver to increase prothrombotic risk. Surgical patients typically experienced blood stasis, endothelial damage and inflammation perioperatively. These predisposed clinical conditions were responsible for thrombin generation, release of vascular cell-derived microparticles and upregulation of platelet adherence molecules, all of which induced platelets activation [32]. As found in our study, a substantial proportion of patients were haunted by platelet-relevant hypercoagulability at the early period after lung cancer surgery. It is noticed that curative resections have removed tumor, therefore, provoked platelets should be blamed on the surgical intervene that introduces direct impact on blood system, which exactly reflects the early alteration of hypercoagulation patterns.

Most international guidelines recommended the use of prophylaxis dose of LMWH in patients undergoing cancer surgery to prevent postoperative VTE [33–35]. Prevention strategy involving LMWH was illustrated to reduce the occurrence of VTE among ambulatory patients with lung cancer [36]. However, a randomized trial discovered significantly differences of individual sensitivity of heparin and suggested that a fixed dosage of heparin administration might not provide sufficient anticoagulation for thromboprophylaxis postoperatively [16]. Even receiving LMWH thromboprophylaxis, some patients still develop VTE [11]. In a recent meta-analysis, it showed that for surgical patients, the benefit of VTE chemoprophylaxis was found only among specific group of patients [37]. In our PSM analysis, the small gaps of hypercoagulable state between patients with and without prophylactic administration of LMWH also indicated its incompetence to alleviate prothrombotic stress. Surgery has extended influence on coagulation function and integrity of vascular endothelial cell. Normally, injury to vascular wall during surgery exposes collagen and then triggers platelet activation to reconstruct vascular integrity, but meanwhile, subsequent secretion of platelet factor 4 from activated platelets has a strong binding ability to heparin, which could repress the heparin dependent anticoagulation with antithrombin III [38]. Even LMWH primary inhibits coagulation factor Xa, it still has extended effects on other coagulation factors in the coagulation cascade, which can affect subsequent platelet activation. But in our study, TEG results suggested that activated platelet still dampened the thromboprophylaxis efficacy of LMWH. Further explanation about the mechanism that activated platelet interrupted the anticoagulation effect of LMWH should be addressed. But before that, it was proclaimed that the platelet activation in clot formation need to be aggressively guided by TEG during prophylactic treatment [39].

In the LMWH group, the number of hypercoagulable patients increased from 9 to 10 following surgery (Table 3), but there were higher proportion of platelet-associated hypercoagulation in hypercoagulable patients at the third day (8/10) compared to that at first day (2/9) after surgery, in the meantime, the proportion of enzymatic hypercoagulation dropped. Even the sample size was small, it was conjectured that even enzymatic hypercoagulation still existed in some patients after anticoagulation, it was useful in reducing hazard of the enzymatic hypercoagulation in clot formation on TEG. Normally, the dubious anticoagulation effectiveness that reflected the insufficiency of prophylaxis can be indicative of higher dose of LMWH [12]. However, the emerging of elevated proportion of platelet hypercoagulation may indicate a lack of thromboprophylaxis by solely adopting anti-thrombogenesis strategy, whereupon the hypercoagulative state sustains [40]. Currently, anti-platelet agents were mostly utilized in preventing artery embolization, venous thromboprophylaxis by means of anti-platelet strategy like aspirin was currently applied only for orthopaedic surgery. As for cancer patients, the use of LMWH represents the current standard prevention for VTE, whereas aspirin remained controversial and lacked for robust evidence for clinical application to reduce thrombotic risk [41]. Even some new anti-platelet agent targeting different platelet ligands was found to effectively inhibit platelet activity without significantly increasing hemorrhage tendency [42, 43], the bleeding risk was still the essential factor that impeded further utilization of anti-platelet in VTE prevention. Overall, viewing the dynamic pattern of hypercoagulable status after lung cancer surgery, we thought that current anticoagulation protocol by mere LMWH was seemingly insufficient, and the combination of anti-platelet therapy may be of additional value for reducing the prothrombotic stress among surgical patients.

Current study has certain limitations that hindered some conclusive interpretations. First, we were unable to investigate the relationship between hypercoagulability and LMWH administration by a randomized method, but we applied PMS to narrow the impact of confounders between groups as possible. Mechanical and pharmacological prophylaxis were most important strategies in VTE prevention. In this study, all patients received standard perioperative mechanical prevention by compress stocking and early mobilization, therefore, there was a reasonable perspective that the changes of coagulation status reflected the real effectiveness of chemoprophylactic LMWH. Second, the number of patients was relatively small, and they were primarily among those with early-stage non-small cell lung cancer diagnosed as adenocarcinoma, which may introduce some biases. Finally, the lack of prolonged monitoring of TEG is another limitation, and yet current results still uncovered significant variation of hypercoagulability based on limited period of monitoring.

## Conclusions

This study tested that TEG results disclosed significantly different hypercoagulative states at different time points in the early period postoperatively, in which the role of platelets cannot be neglected. Also, TEG results found no distinguishably alleviated hypercoagulability in patient with postoperative prophylactic LMWH, which indicated insufficient thromboprophylaxis and warranted extra efforts in determine more efficient VTE prevention strategy. Even more studies based on prospective protocol among larger cohorts are needed, our results were promising to indicate that TEG is an effective tool to stratify various prothrombotic risk at different time points. By incorporating full panel of parameters, TEG can be conductive to monitoring of anticoagulation efficacy when developing proper thromboprophylaxis strategy in further investigations.

## Supplementary Information

Below is the link to the electronic supplementary material.

Supplementary file1 (DOCX 13 KB)

## Data Availability

The data sets used and analyzed in this study are available from the corresponding author on reasonable request.
