# Supplementary material for: Hypercoagulable state and effect of low-molecular-weight heparin prophylaxis on coagulation after lung cancer resection: results from thrombo-elastography
Source: Gen Thorac Cardiovasc Surg. 2024 Jul 26;73(3):180–9. doi: 10.1007/s11748-024-02062-6 (PMC11829937; doi:10.1007/s11748-024-02062-6)
Supplement: Supplementary file 1 — Supplementary file1 (DOCX 13 KB) [file 11748_2024_2062_MOESM1_ESM.docx]

Supplement 1 The baseline between 37 pairs of patients with and without LWMH prophylaxis by propensity scores matching

| Variable | LWMH prophylaxis | No prophylaxis | *P*-value |
| --- | --- | --- | --- |
| Age (years) | 61.8 ± 7.5 | 64.4 ± 6.2 | 0.161 |
| Gender (male) | 22 (59.5) | 20 (54.1) | 0.639 |
| TNM stage |  |  | 0.790 |
| 0 + Ⅰ | 28 (75.7) | 27 (73.0) |  |
| Ⅱ + III | 5 (41.7) | 36 (18.5) |  |
| Postoperative hospitalization (days) | 5 (4-7.5) | 5 (4-7) | 0.591 |
| Surgical approach |  |  | 0.722 |
| Thoracotomy | 4 (10.8) | 5 (13.5) |  |
| VATS | 33 (89.2) | 32 (86.5) |  |
| Duration of operation (mins) | 145.0±65.5 | 123.4±45.4 | 0.122 |

VATS, video-assisted thoracoscopic surgery
